# Supplementary material for: Microbial communities of Schisandra sphenanthera Rehd. et Wils. and the correlations between microbial community and the active secondary metabolites
Source: PeerJ. 2024 Apr 26;12:e17240. doi: 10.7717/peerj.17240 (PMC11057425; doi:10.7717/peerj.17240)
Supplement: Supplemental Information 1 [file peerj-12-17240-s001.doc]

**Supplementary Data**

**Microbial communities of *Schisandra sphenanthera* Rehd. et Wils. and the correlations between microbial community and the active secondary metabolites**

Xiao Lu Qin, Han Pu, Xi Lin Fang, Qian Qian Shang, Jian Hua Li, Qiao Zhu Zhao, Xiao Rui Wang* and Wei Gu*

National Engineering Laboratory for Resource Development of Endangered Crude Drugs in Northwest China, The Key Laboratory of Medicinal Resources and Natural Pharmaceutical Chemistry, The Ministry of Education, College of Life Sciences, Shaanxi Normal University, Xi’an, Shaanxi 710119, P. R. China

* Corresponding Author:

Xiao Rui Wang, Wei Gu

620 West Chang 'an Avenue, Xi’an, Shaanxi 710119, P. R. China

Email address: [xiaoruiwang@snnu.edu.cn](mailto:xiaoruiwang@snnu.edu.cn) (X.W.); [weigu@snnu.edu.cn](mailto:weigu@snnu.edu.cn) (W.G.)

**Table S1.** Sequencing regions and PCR primers are selected for this study.

| Region | Universal primers | Direction | Primers |
| --- | --- | --- | --- |
| 16S V4 | 515F | Forward | 5' GTGYCAGCMGCCGCGGTAA 3' |
|  | 806R | Reverse | 5' GGACTACNVGGGTWTCTAA 3' |
| ITS1 | ITS5-1737F | Forward | 5' GGAAGTAAAAGTCGTAACAAGG 3' |
|  | ITS2-2043R | Reverse | 5' GCTGCGTTCTTCATCGATGC3 ' |

**Table S2.** Factors and levels for the orthogonal test.

| Level (**Stem**) | Factor | | | |
| --- | --- | --- | --- | --- |
|  | A (g/mL) | B (min) | C (°C) | D (W) |
| 1 | 1：10 | 20 | 30 | 240 |
| 2 | 1：15 | 30 | 35 | 270 |
| 3 | 1：20 | 40 | 40 | 300 |
| Level (**Leaf**) | Factor | | | |
|  | A (g/mL) | B (min) | C (°C) | D (W) |
| 1 | 1：15 | 10 | 30 | 210 |
| 2 | 1：20 | 20 | 35 | 240 |
| 3 | 1：25 | 30 | 40 | 270 |

Note A: Ratio of material to solvent; B: Ultrasonic time; C: Ultrasonic temperature; D: Ultrasonic power.

**Table S3.** Results of L9 (3^4^) orthogonal test.

| Number (**Stem**) | Factor | | | | | Extraction rate (%) |
| --- | --- | --- | --- | --- | --- | --- |
|  | A (g/mL) | | B (min) | C (°C) | D (W) |  |
| 1 | 1 | | 1 | 1 | 1 | 0.71±0.13 |
| 2 | 1 | | 2 | 2 | 2 | 1.93±0.22 |
| 3 | 1 | | 3 | 3 | 3 | 0.53±0.15 |
| 4 | 2 | | 1 | 2 | 3 | 3.37±0.47 |
| 5 | 2 | | 2 | 3 | 1 | 3.98±0.11 |
| 6 | 2 | | 3 | 1 | 2 | 0.68±0.21 |
| 7 | 3 | | 1 | 3 | 2 | 3.72±0.18 |
| 8 | 3 | | 2 | 1 | 3 | 4.50±0.22 |
| 9 | 3 | | 3 | 2 | 1 | 1.55±0.19 |
| k1 | 3.17 | | 7.80 | 5.89 | 6.24 |  |
| k2 | 8.03 | | 10.41 | 6.85 | 6.33 |  |
| k3 | 9.77 | | 2.76 | 8.23 | 8.40 |  |
| K1 | 1.06 | | 2.60 | 1.96 | 2.08 |  |
| K2 | 2.68 | | 3.47 | 2.28 | 2.11 |  |
| K3 | 3.26 | | 0.92 | 2.74 | 2.80 |  |
| R | 2.20 | | 2.55 | 0.78 | 0.72 |  |
| Factor sequence | B>A>C>D | | | | | |
| Optimal combination | | A_3_B_2_C_1_D_3_ | | | | |
| Number (**Leaf**) | Factor | | | | | Extraction rate (%) |
|  | A (g/mL) | | B (min) | C (°C) | D (W) |  |
| 1 | 1 | | 1 | 1 | 1 | 5.03±0.15 |
| 2 | 1 | | 2 | 2 | 2 | 5.16±0.67 |
| 3 | 1 | | 3 | 3 | 3 | 3.67±0.91 |
| 4 | 2 | | 1 | 2 | 3 | 4.80±0.25 |
| 5 | 2 | | 2 | 3 | 1 | 4.19±0.32 |
| 6 | 2 | | 3 | 1 | 2 | 3.80±0.64 |
| 7 | 3 | | 1 | 3 | 2 | 3.81±0.09 |
| 8 | 3 | | 2 | 1 | 3 | 2.74±0.27 |
| 9 | 3 | | 3 | 2 | 1 | 2.91±0.35 |
| k1 | 13.86 | | 13.64 | 11.57 | 12.13 |  |
| k2 | 12.79 | | 12.09 | 12.87 | 12.77 |  |
| k3 | 9.46 | | 10.38 | 11.67 | 11.21 |  |
| K1 | 4.62 | | 4.55 | 3.86 | 4.04 |  |
| K2 | 4.26 | | 4.03 | 4.29 | 4.26 |  |
| K3 | 3.15 | | 3.46 | 3.89 | 3.74 |  |
| R | 1.47 | | 1.09 | 0.43 | 0.52 |  |
| Factor sequence | A>B>D>C | | | | | |
| Optimal combination | | A_1_B_2_C_2_D_2_ | | | | |

Note SD: standard deviation; A: Ratio of material to solvent; B: Ultrasonic time; C: Ultrasonic temperature; D: Ultrasonic power.

**Table S4.** Table of variance analysis of the orthogonal test.

| Factor (**Stem**) | Sum of Squares | Degree of freedom | Mean square | F | Significance |
| --- | --- | --- | --- | --- | --- |
| A | 23.377 | 2 | 11.688 | 103.862 | ** |
| B | 29.850 | 2 | 14.925 | 132.623 | ** |
| C | 2.724 | 2 | 1.362 | 12.103 | ** |
| D | 2.971 | 2 | 1.485 | 13.200 | ** |
| error | 2.026 |  | 0.113 |  |  |
| sum | 207.858 |  |  |  |  |
| Factor (**Leaf**) | Sum of Squares | Degree of freedom | Mean square | F | Significance |
| A | 10.440 | 2 | 5.220 | 22.607 | ** |
| B | 5.303 | 2 | 2.652 | 11.483 | ** |
| C | 1.096 | 2 | 0.548 | 2.374 |  |
| D | 1.222 | 2 | 0.611 | 2.626 | * |
| error | 4.156 |  | 0.231 |  |  |
| sum | 457.012 |  |  |  |  |

Note A: Ratio of material to solvent; B: Ultrasonic time; C: Ultrasonic temperature; D: Ultrasonic power; *: *p* < 0.05; **: *p* < 0.01.

**Table S5. Level of bacterial microorganism annotation abundances table (the level of genus).**

| No.* | Genus | Rhizosphere soil | Root | Stem | Leaf | Fruit |
| --- | --- | --- | --- | --- | --- | --- |
| 1 | *Afifella* | 0.059±0.022 | 0.274±0.127 | 0.000±0.000 | 0.000±0.000 | 0.000±0.000 |
| 2 | *Candidatus Solibacter* | 1.410±0.512 | 0.362±0.138 | 0.000±0.000 | 0.000±0.000 | 0.000±0.000 |
| 3 | *Chitinophaga* | 0.029±0.011 | 0.563±0.184 | 0.000±0.000 | 0.000±0.000 | 0.000±0.000 |
| 4 | *Dokdonella* | 0.238±0.048 | 0.168±0.038 | 0.000±0.000 | 0.000±0.000 | 0.000±0.000 |
| 5 | *Inquilinus* | 0.214±0.087 | 0.052±0.008 | 0.000±0.000 | 0.000±0.000 | 0.000±0.000 |
| 6 | *Nitrospira* | 0.178±0.074 | 0.037±0.007 | 0.000±0.000 | 0.000±0.000 | 0.000±0.000 |
| 7 | *Novosphingobium* | 0.006±0.001 | 0.274±0.075 | 0.000±0.000 | 0.000±0.000 | 0.000±0.000 |
| 8 | *Opitutus* | 0.009±0.003 | 0.206±0.086 | 0.000±0.000 | 0.000±0.000 | 0.000±0.000 |
| 9 | *Paucibacter* | 0.163±0.054 | 0.197±0.088 | 0.000±0.000 | 0.000±0.000 | 0.000±0.000 |
| 10 | *Pedomicrobium* | 0.502±0.217 | 0.173±0.094 | 0.000±0.000 | 0.000±0.000 | 0.000±0.000 |
| 11 | *Rhizobium* | 0.443±0.148 | 0.378±0.315 | 0.000±0.000 | 0.000±0.000 | 0.000±0.000 |
| 12 | *Rhodoplanes* | 2.209±1.029 | 4.280±2.127 | 0.000±0.000 | 0.000±0.000 | 0.000±0.000 |
| 13 | *Bacteroides* | 0.000±0.000 | 0.000±0.000 | 0.330±0.169 | 0.517±0.133 | 0.161±0.107 |
| 14 | *Brevibacillus* | 0.000±0.000 | 0.000±0.000 | 0.245±0.117 | 0.024±0.011 | 0.046±0.018 |
| 15 | *Blastomonas* | 0.000±0.000 | 0.000±0.000 | 0.265±0.099 | 0.000±0.000 | 0.053±0.019 |
| 16 | *Cloacibacterium* | 0.000±0.000 | 0.000±0.000 | 0.154±0.067 | 0.594±0.226 | 0.000±0.000 |
| 17 | *Corynebacterium* | 0.000±0.000 | 0.000±0.000 | 0.455±0.208 | 0.330±0.124 | 0.168±0.066 |
| 18 | *Deinococcus* | 0.000±0.000 | 0.000±0.000 | 0.266±0.135 | 0.000±0.000 | 0.136±0.062 |
| 19 | *Fusobacterium* | 0.000±0.000 | 0.000±0.000 | 0.079±0.033 | 0.177±0.033 | 0.037±0.021 |
| 20 | *Haliscomenobacter* | 0.000±0.000 | 0.000±0.000 | 0.000±0.000 | 0.223±0.182 | 0.000±0.000 |
| 21 | *Hydrocarboniphaga* | 0.000±0.000 | 0.000±0.000 | 0.000±0.000 | 0.000±0.000 | 0.197±0.038 |
| 22 | *Jeotgalicoccus* | 0.000±0.000 | 0.000±0.000 | 0.000±0.000 | 0.140±0.088 | 0.000±0.000 |
| 23 | *Limnobacter* | 0.000±0.000 | 0.000±0.000 | 0.167±0.088 | 0.103±0.067 | 0.213±0.101 |
| 24 | *Micrococcus* | 0.000±0.000 | 0.000±0.000 | 0.514±0.149 | 0.107±0.067 | 0.406±0.201 |
| 25 | *Parabacteroides* | 0.000±0.000 | 0.000±0.000 | 0.000±0.000 | 0.263±0.104 | 0.000±0.000 |
| 26 | *Paracoccus* | 0.000±0.000 | 0.000±0.000 | 0.327±0.138 | 0.000±0.000 | 0.058±0.027 |
| 27 | *Pigmentiphaga* | 0.000±0.000 | 0.000±0.000 | 0.264±0.113 | 0.103±0.010 | 0.000±0.000 |
| 28 | *Prevotella* | 0.000±0.000 | 0.000±0.000 | 0.081±0.021 | 0.317±0.163 | 0.037±0.011 |
| 29 | *Anoxybacillus* | 0.000±0.000 | 0.046±0.021 | 4.438±1.328 | 2.230±1.322 | 1.088±0.827 |
| 30 | *Bacillus* | 0.000±0.000 | 0.033±0.011 | 0.606±0.286 | 0.925±0.341 | 0.461±0.303 |
| 31 | *Brevundimonas* | 0.000±0.000 | 0.006±0.003 | 0.145±0.061 | 0.000±0.000 | 0.000±0.000 |
| 32 | *Chryseobacterium* | 0.000±0.000 | 0.023±0.011 | 0.147±0.034 | 0.111±0.009 | 0.183±0.019 |
| 33 | *Curtobacterium* | 0.000±0.000 | 0.012±0.005 | 0.771±0.118 | 0.385±0.113 | 0.317±0.108 |
| 34 | *Friedmanniella* | 0.000±0.000 | 0.017±0.007 | 0.105±0.011 | 0.178±0.018 | 0.227±0.039 |
| 35 | *Kocuria* | 0.000±0.000 | 0.004±0.001 | 0.298±0.102 | 0.000±0.000 | 0.002±0.001 |
| 36 | *Methyloversatilis* | 0.000±0.000 | 0.066±0.009 | 1.669±0.889 | 0.409±0.207 | 1.767±0.862 |
| 37 | *Patulibacter* | 0.000±0.000 | 0.012±0.002 | 0.000±0.000 | 0.195±0.088 | 0.053±0.027 |
| 38 | *Staphylococcus* | 0.000±0.000 | 0.012±0.001 | 5.901±2.357 | 0.916±0.233 | 0.526±0.208 |
| 39 | *Thermus* | 0.000±0.000 | 0.012±0.004 | 0.366±0.122 | 0.000±0.000 | 0.408±0.107 |
| 40 | *Aeromicrobium* | 0.002±0.000 | 0.000±0.000 | 0.151±0.077 | 0.000±0.000 | 0.011±0.008 |
| 41 | *Agrobacterium* | 0.077±0.019 | 0.875±0.237 | 0.357±0.119 | 0.000±0.000 | 0.264±0.117 |
| 42 | *Caulobacter* | 0.063±0.027 | 0.179±0.069 | 0.000±0.000 | 0.901±0.332 | 0.275±0.079 |
| 43 | *Devosia* | 0.059±0.019 | 0.253±0.113 | 0.000±0.000 | 0.000±0.000 | 0.096±0.034 |
| 44 | *Frigoribacterium* | 0.002±0.001 | 0.004±0.002 | 0.156±0.088 | 0.000±0.000 | 0.512±0.224 |
| 45 | *Gemmata* | 1.067±0.754 | 0.513±0.201 | 0.681±0.223 | 0.000±0.000 | 0.261±0.043 |
| 46 | *Hymenobacter* | 0.017±0.008 | 0.000±0.000 | 0.295±0.101 | 2.210±1.087 | 0.564±0.201 |
| 47 | *Luteibacter* | 0.203±0.057 | 0.013±0.003 | 0.000±0.000 | 0.174±0.082 | 0.000±0.000 |
| 48 | *Lysobacter* | 0.098±0.037 | 0.000±0.000 | 0.203±0.103 | 0.165±0.088 | 0.473±0.161 |
| 49 | *Microbacterium* | 0.003±0.001 | 0.015±0.007 | 0.311±0.118 | 0.000±0.000 | 0.050±0.011 |
| 50 | *Paenibacillus* | 0.008±0.003 | 0.000±0.000 | 0.552±0.207 | 0.428±0.201 | 0.138±0.093 |
| 51 | *Pseudonocardia* | 0.017±0.005 | 0.256±0.102 | 0.000±0.000 | 0.000±0.000 | 0.019±0.007 |
| 52 | *Sphingobium* | 0.019±0.008 | 0.320±0.132 | 0.000±0.000 | 0.000±0.000 | 0.048±0.013 |
| 53 | *Spirosoma* | 0.091±0.013 | 0.013±0.007 | 0.000±0.000 | 0.601±0.104 | 0.184±0.082 |
| 54 | *Streptomyces* | 0.014±0.007 | 0.426±0.032 | 0.253±0.023 | 0.000±0.000 | 0.000±0.000 |
| 55 | *others* | 6.671±1.257 | 1.629±0.731 | 0.929±0.127 | 0.419±0.224 | 0.929±0.337 |

* The genera counted in the table are OTUs more than 100 (at least one part of *S. sphenanthera*). 1-12: Bacterial microorganisms exist only in rhizosphere soil and roots. 13-28: Bacterial microorganisms are present in the above-ground part (stem, leaf, and fruit). 29-39: Bacterial microorganisms are present in the root, stem, leaf, and fruit. 40-55: The bacterial microorganism with no apparent distribution pattern.

**Table S6. Level of fungal microorganism annotation abundances table (the level of genus).**

| No.* | Genus | Rhizosphere soil | Root | Stem | Leaf | Fruit |
| --- | --- | --- | --- | --- | --- | --- |
| 1 | *Chaetosphaeria* | 0.169±0.011 | 0.000±0.000 | 0.000±0.000 | 0.000±0.000 | 0.000±0.000 |
| 2 | *Clavulinopsis* | 2.101±0.776 | 0.000±0.000 | 0.000±0.000 | 0.000±0.000 | 0.000±0.000 |
| 3 | *Exophiala* | 0.208±0.092 | 0.000±0.000 | 0.000±0.000 | 0.000±0.000 | 0.000±0.000 |
| 4 | *Hebeloma* | 10.830±2.338 | 0.000±0.000 | 0.000±0.000 | 0.000±0.000 | 0.000±0.000 |
| 5 | *Hymenogaster* | 0.953±0.213 | 0.000±0.000 | 0.000±0.000 | 0.000±0.000 | 0.000±0.000 |
| 6 | *Striaticonidium* | 0.518±0.288 | 0.000±0.000 | 0.000±0.000 | 0.000±0.000 | 0.000±0.000 |
| 7 | *Entoloma* | 0.282±0.107 | 0.136±0.089 | 0.000±0.000 | 0.000±0.000 | 0.000±0.000 |
| 8 | *Glomus* | 0.025±0.010 | 0.651±0.207 | 0.000±0.000 | 0.000±0.000 | 0.000±0.000 |
| 9 | *Leptonia* | 0.203±0.113 | 0.197±0.008 | 0.000±0.000 | 0.000±0.000 | 0.000±0.000 |
| 10 | *Trichoderma* | 0.008±0.001 | 0.214±0.103 | 0.000±0.000 | 0.000±0.000 | 0.000±0.000 |
| 11 | *Tuber* | 1.176±0.872 | 0.678±0.324 | 0.000±0.000 | 0.000±0.000 | 0.000±0.000 |
| 12 | *unclassified_*  *Clavariaceae* | 1.887±0.934 | 0.898±0.227 | 0.000±0.000 | 0.000±0.000 | 0.000±0.000 |
| 13 | *unclassified_*  *Glomeraceae* | 0.032±0.011 | 0.439±0.115 | 0.000±0.000 | 0.000±0.000 | 0.000±0.000 |
| 14 | *Auricularia* | 0.000±0.000 | 0.000±0.000 | 0.031±0.017 | 0.223±0.107 | 0.000±0.000 |
| 15 | *Catenulostroma* | 0.000±0.000 | 0.000±0.000 | 0.000±0.000 | 0.238±0.099 | 0.000±0.000 |
| 16 | *Derxomyces* | 0.000±0.000 | 0.000±0.000 | 0.000±0.000 | 0.157±0.082 | 0.000±0.000 |
| 17 | *Eremothecium* | 0.000±0.000 | 0.000±0.000 | 0.000±0.000 | 0.000±0.000 | 1.150±0.883 |
| 18 | *Glomerella* | 0.000±0.000 | 0.000±0.000 | 0.000±0.000 | 0.150±0.071 | 0.000±0.000 |
| 19 | *Microascus* | 0.000±0.000 | 0.000±0.000 | 0.000±0.000 | 0.000±0.000 | 1.838±1.021 |
| 20 | *Microcyclosporella* | 0.000±0.000 | 0.000±0.000 | 0.282±0.077 | 0.866±0.233 | 0.284±0.091 |
| 21 | *Paraconiothyrium* | 0.000±0.000 | 0.000±0.000 | 0.000±0.000 | 0.242±0.137 | 0.021±0.008 |
| 22 | *Peyronellaea* | 0.000±0.000 | 0.000±0.000 | 0.221±0.101 | 0.137±0.006 | 0.133±0.007 |
| 23 | *Seimatosporium* | 0.000±0.000 | 0.000±0.000 | 0.092±0.011 | 0.343±0.109 | 0.000±0.000 |
| 24 | *Stomiopeltis* | 0.000±0.000 | 0.000±0.000 | 18.506±5.333 | 0.477±0.119 | 1.173±0.537 |
| 25 | *unclassified_*  *Bionectriaceae* | 0.000±0.000 | 0.000±0.000 | 0.245±0.100 | 0.000±0.000 | 0.000±0.000 |
| 26 | *unclassified_*  *Thelebolaceae* | 0.000±0.000 | 0.000±0.000 | 0.247±0.118 | 0.000±0.000 | 0.000±0.000 |
| 27 | *Uwebraunia* | 0.000±0.000 | 0.000±0.000 | 0.646±0.209 | 0.288±0.104 | 1.066±0.428 |
| 28 | *Vishniacozyma* | 0.000±0.000 | 0.000±0.000 | 0.000±0.000 | 0.079±0.037 | 0.388±0.107 |
| 29 | *Zygophiala* | 0.000±0.000 | 0.000±0.000 | 0.017±0.007 | 0.058±0.021 | 5.657±2.119 |
| 30 | *Fusarium* | 0.000±0.000 | 0.214±0.107 | 1.689±0.758 | 0.132±0.067 | 0.188±0.071 |
| 31 | *Malassezia* | 0.000±0.000 | 0.178±0.088 | 0.028±0.014 | 0.027±0.011 | 0.034±0.013 |
| 32 | *Naganishia* | 0.000±0.000 | 0.562±0.231 | 0.000±0.000 | 0.633±0.227 | 0.000±0.000 |
| 33 | *Phlebia* | 0.000±0.000 | 0.121±0.082 | 0.807±0.237 | 0.116±0.052 | 0.031±0.012 |
| 34 | *Scleroramularia* | 0.000±0.000 | 0.047±0.021 | 1.401±0.352 | 0.368±0.114 | 1.418±0.237 |
| 35 | *Trametes* | 0.000±0.000 | 0.067±0.017 | 1.430±0.371 | 0.040±0.014 | 0.065±0.021 |
| 36 | *unclassified_*  *Eurotiales* | 0.000±0.000 | 0.905±0.320 | 0.033±0.011 | 0.010±0.006 | 0.034±0.013 |
| 37 | *unclassified_*  *Hysteriaceae* | 0.000±0.000 | 0.220±0.103 | 0.011±0.002 | 0.062±0.011 | 0.065±0.023 |
| 38 | *unclassified_*  *Peniophoraceae* | 0.000±0.000 | 0.358±0.112 | 0.111±0.008 | 0.012±0.007 | 0.016±0.007 |
| 39 | *Botrytis* | 0.009±0.002 | 0.000±0.000 | 0.000±0.000 | 0.117±0.027 | 20.768±5.337 |
| 40 | *Chaetomium* | 0.016±0.008 | 0.084±0.031 | 0.236±0.073 | 0.024±0.017 | 0.000±0.000 |
| 41 | *Cortinarius* | 7.498±2.377 | 0.000±0.000 | 0.000±0.000 | 0.000±0.000 | 0.050±0.033 |
| 42 | *Cyphellophora* | 0.052±0.023 | 0.002±0.001 | 0.000±0.000 | 1.602±0.384 | 0.490±0.219 |
| 43 | *Mortierella* | 0.522±0.207 | 0.093±0.039 | 0.266±0.101 | 0.000±0.000 | 0.000±0.000 |
| 44 | *Myrothecium* | 0.020±0.010 | 0.000±0.000 | 0.244±0.116 | 0.126±0.028 | 0.047±0.021 |
| 45 | *Paraphoma* | 0.002±0.001 | 0.000±0.000 | 0.013±0.004 | 0.826±0.392 | 0.000±0.000 |
| 46 | *Pestalotiopsis* | 0.061±0.021 | 0.000±0.000 | 0.279±0.113 | 0.627±0.201 | 0.235±0.102 |
| 47 | *Plectosphaerella* | 0.002±0.000 | 0.000±0.000 | 0.037±0.012 | 0.200±0.034 | 0.010±0.006 |
| 48 | *Ramularia* | 0.009±0.002 | 0.000±0.000 | 0.142±0.068 | 3.544±1.037 | 2.815±0.769 |
| 49 | *Sarocladium* | 0.003±0.001 | 0.000±0.000 | 0.000±0.000 | 0.000±0.000 | 0.605±0.116 |
| 50 | *unclassified_*  *Agaricales* | 5.444±2.137 | 3.123±1.013 | 0.000±0.000 | 0.015±0.004 | 0.000±0.000 |
| 51 | *unclassified_*  *Chaetothyriales* | 0.109±0.022 | 0.002±0.000 | 0.000±0.000 | 1.374±0.201 | 0.086±0.033 |
| 52 | *unclassified_*  *Helotiales* | 0.712±0.116 | 11.790±2.377 | 0.000±0.000 | 0.156±0.049 | 0.099±0.021 |
| 53 | *unclassified_*  *Lasiosphaeriaceae* | 0.282±0.107 | 0.000±0.000 | 0.257±0.116 | 0.000±0.000 | 0.000±0.000 |
| 54 | *unclassified_*  *Orbiliomycetes* | 0.241±0.094 | 0.000±0.000 | 0.024±0.011 | 0.000±0.000 | 0.000±0.000 |
| 55 | *unclassified_*  *Pleosporales* | 0.044±0.021 | 0.017±0.008 | 0.002±0.000 | 0.665±0.131 | 0.086±0.045 |
| 56 | *unclassified_*  *Sordariomycetes* | 0.101±0.007 | 0.000±0.000 | 1.157±0.852 | 0.030±0.011 | 0.000±0.000 |
| 57 | *unclassified_*  *Thelephoraceae* | 3.758±1.055 | 0.604±0.313 | 0.000±0.000 | 0.083±0.027 | 0.000±0.000 |
| 58 | *unclassified_*  *Xylariales* | 1.511±0.331 | 0.000±0.000 | 0.000±0.000 | 0.018±0.008 | 0.005±0.001 |
| 59 | *other* | 50.009±5.114 | 58.710±4.332 | 6.023±1.025 | 15.804±2.009 | 13.420±3.038 |

* 1-6: Fungal microorganisms exist only in rhizosphere soil. 7-13: Fungal microorganisms exist only in rhizosphere soil and roots. 14-29: Fungal microorganisms are present in the above-ground part (stem, leaf, and fruit). 30-38: Fungal microorganisms are present in the root, stem, leaf, and fruit. 39-59: The fungal microorganism with no apparent distribution pattern.

**Table S7.** Chemical compositions identified in the essential oils of different parts of *S. sphenanthera*.

| No. | Composition | Molecular formula | Stem (%) | Leaf (%) | Fruit (%) | RT | RI |
| --- | --- | --- | --- | --- | --- | --- | --- |
| 1 | *β*-Methylionone | C_14_H_22_O | 0.739±0.19 | 1.017±0.191 | - | 18.764 | 1871 |
| 2 | Isosativene | C_15_H_24_ | 0.463±0.027 | - | 0.323±0.165 | 23.387 | 1417 |
| 3 | *α*.-Amorphene | C_15_H_24_ | 1.618±0.194 a | 1.564±0.217 a | 0.331±0.322 b | 24.253 | 1482 |
| 4 | Germacrene D | C_15_H_24_ | 0.672±0.025 b | 1.497±0.201 a | 0.411±0.022 c | 24.679 | 1515 |
| 5 | *α* -Bergamotene | C_15_H_24_ | - | - | 0.329±0.087 | 25.632 | 1433 |
| 6 | *β* -Santalene | C_15_H_24_ | 0.703±0.054 b | 1.965±0.349 a | 0.503±0.133 b | 25.695 | 1425 |
| 7 | Cuparene | C_15_H_22_ | - | - | 0.199±0.071 | 25.796 | 1511 |
| 8 | Cadinene | C_15_H_24_ | - | 0.616±0.096 | 0.409±0.078 | 26.594 | 1440 |
| 9 | Nerolidol | C_15_H_26_O | 0.462±0.071 | - | 0.223±0.075 | 26.701 | 1535 |
| 10 | *α* -Muurolene | C_15_H_24_ | 0.343±0.014 | - | 0.238±0.021 | 26.904 | 1497 |
| 11 | *α* -Bisabolene | C_15_H_24_ | 0.781±0.045 a | 0.401±0.055 b | 0.760±0.026 a | 27.352 | 1443 |
| 12 | trans*-α*-Bergamotene | C_15_H_24_ | 1.505±0.141 a | 2.185±0.665 a | 0.195±0.116 b | 27.922 | 1477 |
| 13 | *β* -Himachalene | C_15_H_24_ | 0.879±0.000 a | 0.531±0.011 c | 0.805±0.061 b | 28.140 | 1547 |
| 14 | Farnesyl alcohol | C_15_H_26_O | 1.413±0.145 a | 0.847±0.078 b | 0.892±0.259 b | 28.416 | 1713 |
| 15 | *γ* -Muurolene | C_15_H_24_ | **12.381±0.06** a | 3.182±0.881 b | 1.212±0.070 c | 28.536 | 1474 |
| 16 | *δ* -Cadinene | C_15_H_24_ | 2.419±0.904 a | 0.385±0.022 b | 0.350±0.043 b | 28.818 | 1519 |
| 17 | *β-*Bisabolene | C_15_H_24_ | 0.794±0.071 a | 0.281±0.006 b | 0.207±0.043 b | 29.078 | 1506 |
| 18 | Farnesol | C_15_H_26_O | 0.536±0.043 a | 0.570±0.027 a | 0.206±0.057 b | 29.604 | 1416 |
| 19 | trans-Nerolidol | C_15_H_26_O | 3.741±0.205 a | 2.337±0.434 b | 0.705±0.014 c | 30.201 | 1551 |
| 20 | Germacrene D-4-ol | C_15_H_26_O | 3.103±0.019 a | 1.077±0.119 b | 0.677±0.197 c | 30.316 | 1569 |
| 21 | *α* -Limonene diepoxide | C_10_H_16_O_2_ | 4.472±0.071 a | 3.337±0.081 b | 1.299±0.111 c | 30.455 | 1294 |
| 22 | D-nerolidol | C_15_H_26_O | 0.682±0.010 a | 0.613±0.075 a | 0.879±0.383 a | 31.894 | 1532 |
| 23 | *δ* -Cadinol | C_15_H_26_O | **7.739±0.291 a** | 0.820±0.010 b | 0.730±0.121 b | 32.169 | 1652 |
| 24 | *α* -Cadinol | C_15_H_26_O | 3.649±0.151 ab | **6.316±3.210** a | 1.450±0.013 b | 32.551 | 1650 |
| 25 | Dipentene dioxide | C_10_H_16_O_2_ | - | 0.549±0.053 | 1.809±0.171 | 33.225 | 1129 |
| 26 | *α*-Bisabolol | C_15_H_26_O | 1.847±0.071 b | 0.547±0.211 c | 2.175±0.078 a | 33.398 | 1680 |
| 27 | Santalol | C_15_H_24_O | 0.821±0.014 b | 0.217±0.018 c | 1.421±0.361 a | 33.816 | 1617 |
| 28 | trans-Farnesol | C_15_H_26_O | **10.703±0.104** a | 2.337±0.105 b | 1.007±0.065 c | 34.572 | 1722 |
| 29 | Isospathulenol | C_15_H_24_O | - | 0.695±0.319 | **12.428±0.96** | 34.839 | 1582 |
| 30 | *α -*Santalol | C_15_H_24_O | - | 0.508±0.056 | **9.908±0.474** | 35.160 | 1671 |
| 31 | Aromadendrene oxide | C_15_H_24_O | - | 0.419±0.013 | 0.443±0.008 | 35.304 | 1662 |
| 32 | *β* -Elemene | C_15_H_24_ | - | 0.433±0.015 | 0.604±0.046 | 35.403 | 1394 |
| 33 | Cedrenol | C_15_H_24_O | 0.423±0.098 | - | **12.433±1.064** | 35.510 | 1604 |
| 34 | *β* -Chamigrene | C_15_H_24_ | 0.497±0.113 b | 0.397±0.165 b | 2.730±0.460 a | 35.802 | 1478 |
| 35 | Longiverbenone | C_15_H_22_O | - | 0.257±0.046 | **12.723±1.35** | 35.898 | 1651 |
| 36 | Isolongifolen-5-one | C_15_H_22_O | - | 0.282±0.061 | 2.015±1.454 | 36.385 | 1685 |
| 37 | *δ* -Guaiene | C_15_H_24_ | - | 0.306±0.053 | 1.073±0.229 | 36.477 | 1505 |
| 38 | Spathulenol | C_15_H_24_O | - | 0.275±0.073 | 3.275±0.267 | 36.612 | 1536 |
| 39 | Ledene oxide | C_15_H_24_O | 2.631±0.233 a | 0.673±0.213 b | 0.410±0.002 b | 37.199 | 2062 |
| 40 | *β* -Ionone | C_13_H_20_O | - | 0.624±0.077 | 1.644±0.233 | 37.690 | 1485 |
| 41 | Neoisolongifolene-8-ol | C_15_H_24_O | 1.717±0.137 b | **12.779±0.711** a | 0.656±0.297 c | 38.304 | 1578 |
|  | Common chemical compositions |  | 65.303±1.108 | 44.857±3.131 | 20.012±0.551 |  |  |
|  | Total |  | 67.733±1.062 | 50.838±3.137 | 80.088±4.703 |  |  |

The components underlined in the table are common components, and the numbers in bold indicate that the component is the dominant component of the corresponding position. Different lowercase letters (a-c) indicate significant differences (*p* < 0.05) of the common chemical compositions in different parts of *S. sphenanthera*, one-way ANOVA, and Tukey test.


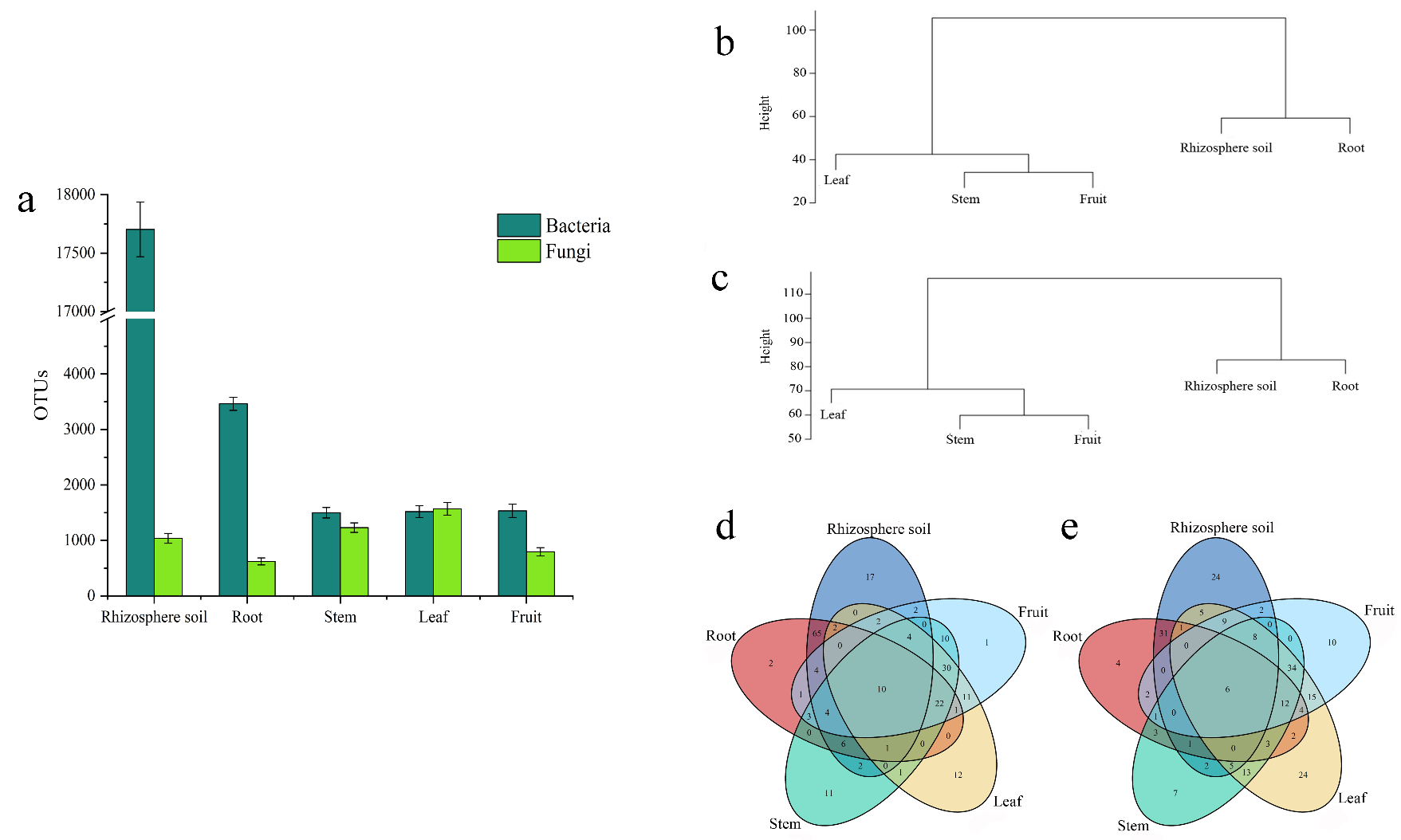


**Fig. S1.** OUTs statistical analysis of microorganisms in rhizosphere soil and different parts of *S. sphenanthera*. a: The number of OTUs, b and c: The cluster dendrogram of identified bacteria and fungi, d (Bacteria) and e (Fungi): Venn diagram analysis on OTUs.


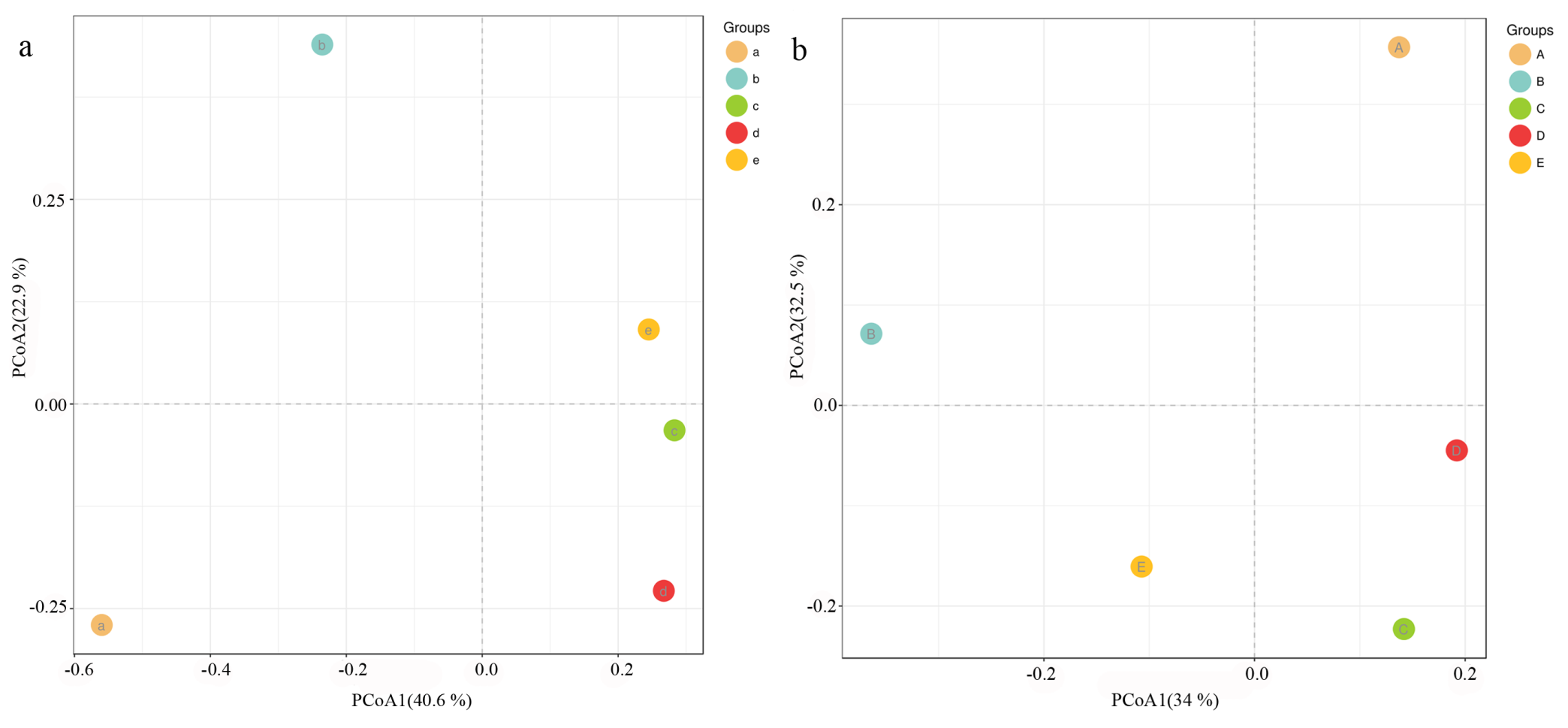


**Fig. S2.** The principal coordinate analysis of rhizosphere soil and different parts of *S. sphenanthera*. a: Bacteria. b: Fungi. a-e and A-E: Rhizosphere soil, Root, Stem, Leaf, and Fruit.


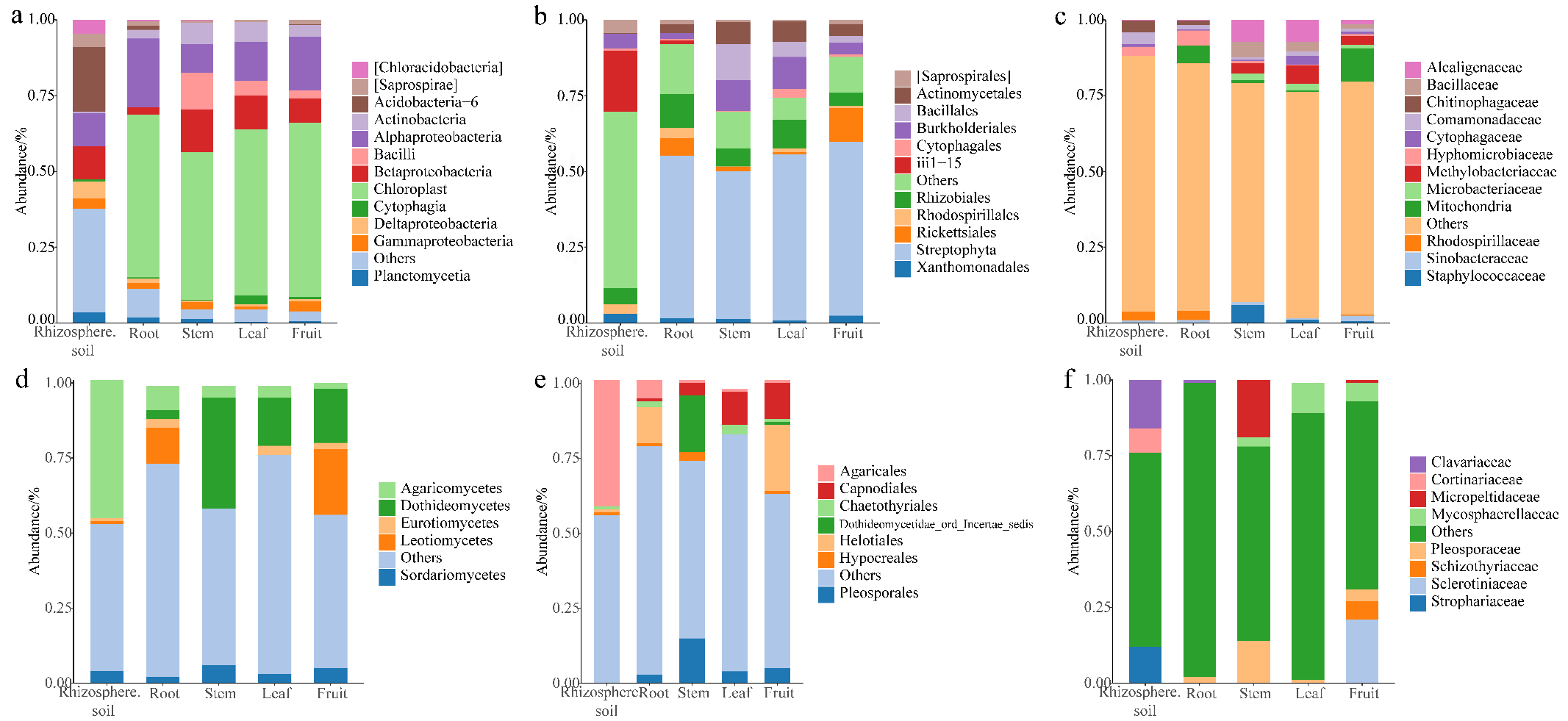


**Fig. S3.** Stack diagram of microorganisms in rhizosphere soil and different parts of *S. sphenanthera*. a-c: Class, Order, and Family of bacteria level. d-f: Class, Order, and Family of fungi level.


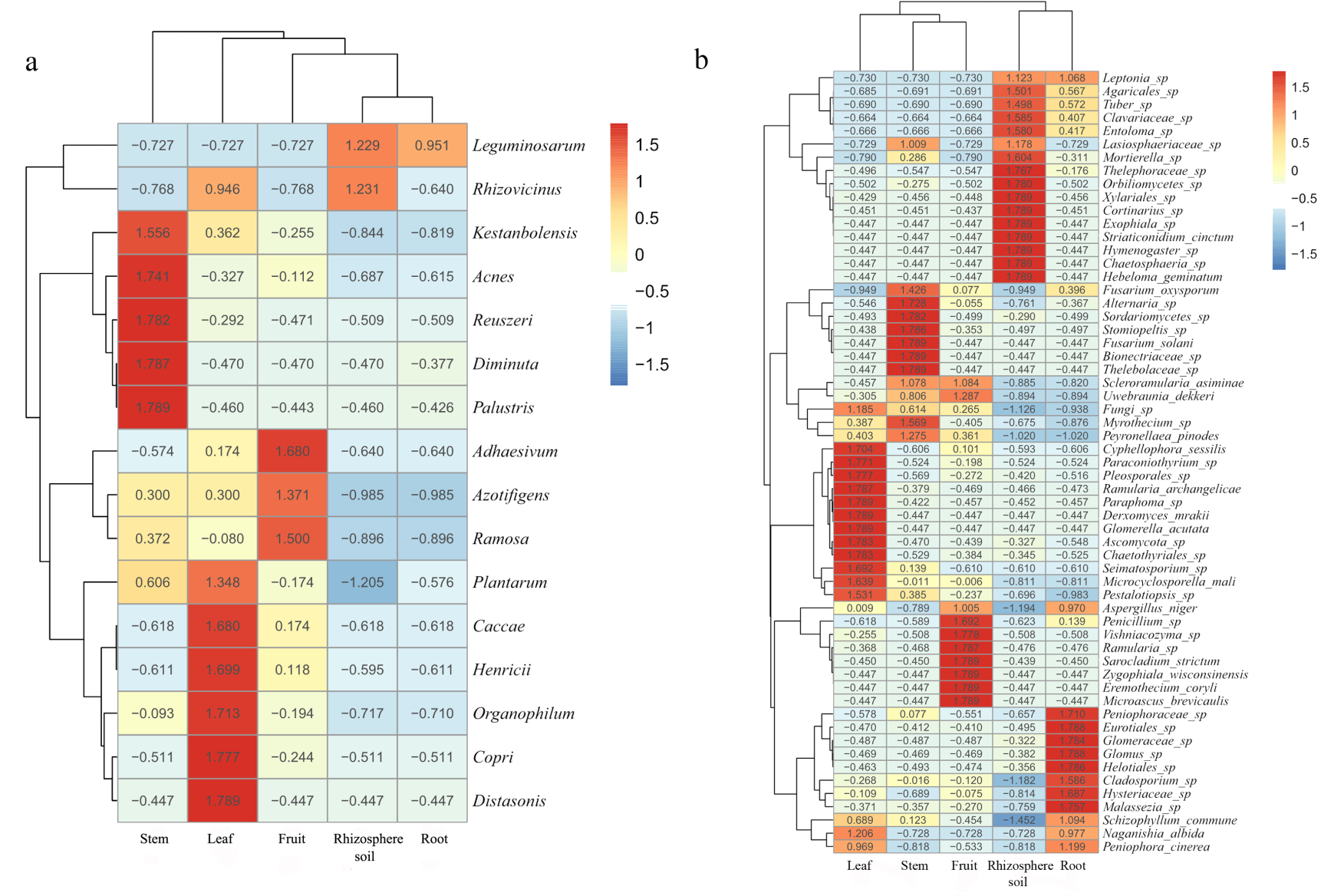


**Fig. S4.** Cluster heat map of relative abundance at the sample’s microbial species level. a: Bacteria. b: Fungi.


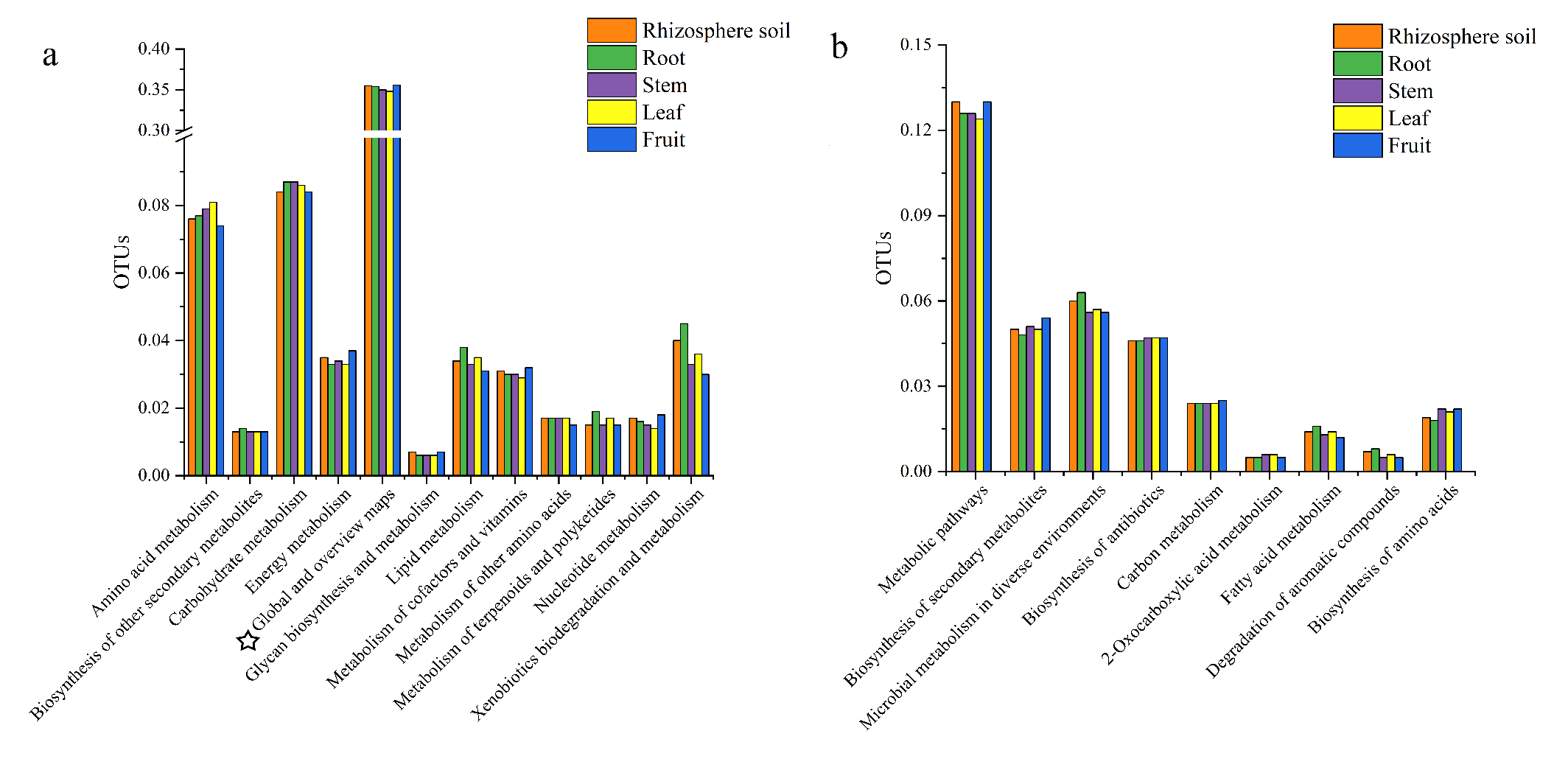


**Fig. S5.** Bacterial function annotation (a: metabolism, b: Global and overview maps) in rhizosphere soil and different parts of *S. sphenanthera*. The function marked by the five-pointed star in Figure a is the content shown in Figure b.


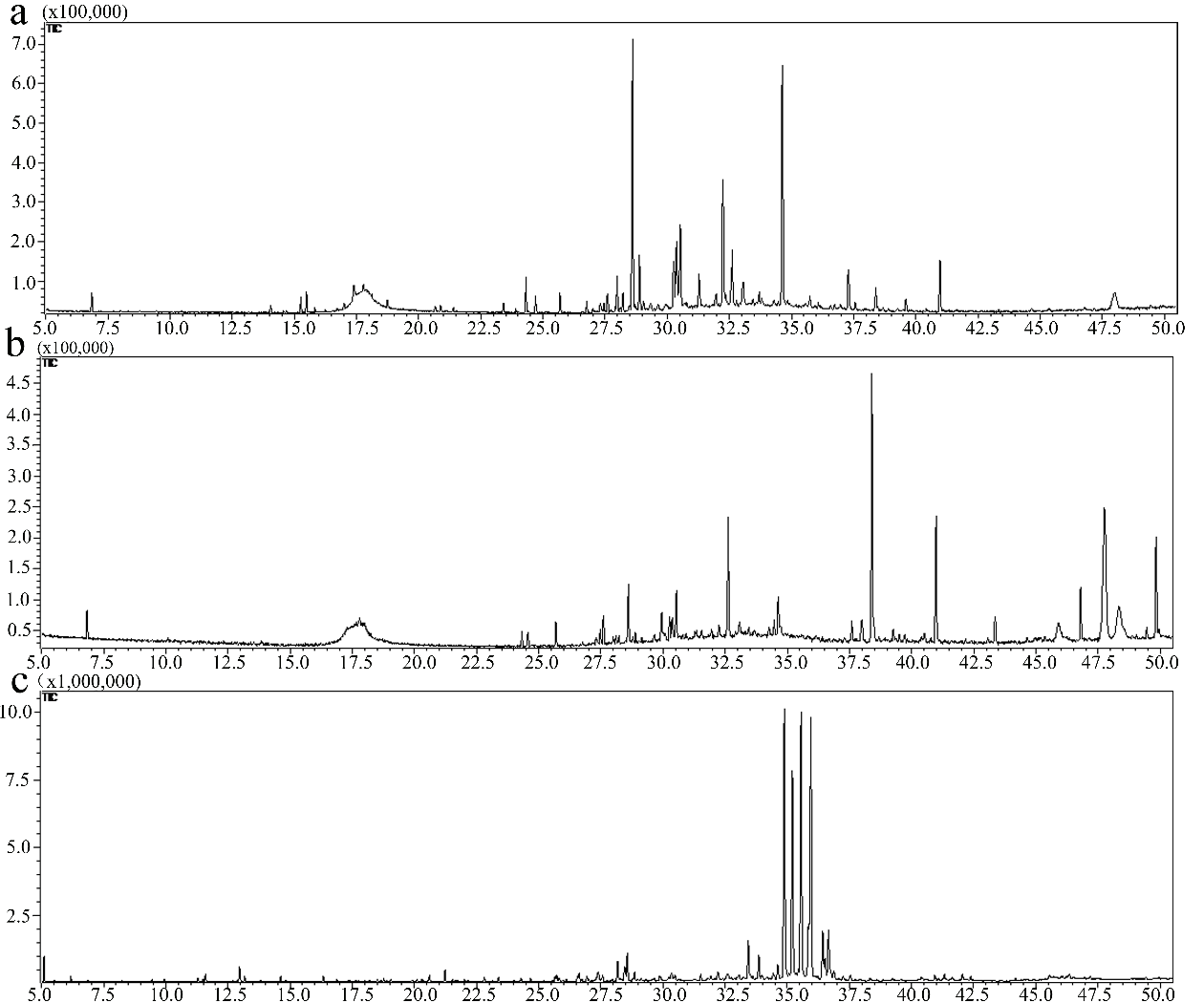


**Fig. S6.** The total ion chromatograms of the stem (A), leaf (B), and fruit (C) of *S. sphenanthera* under GC-MS.


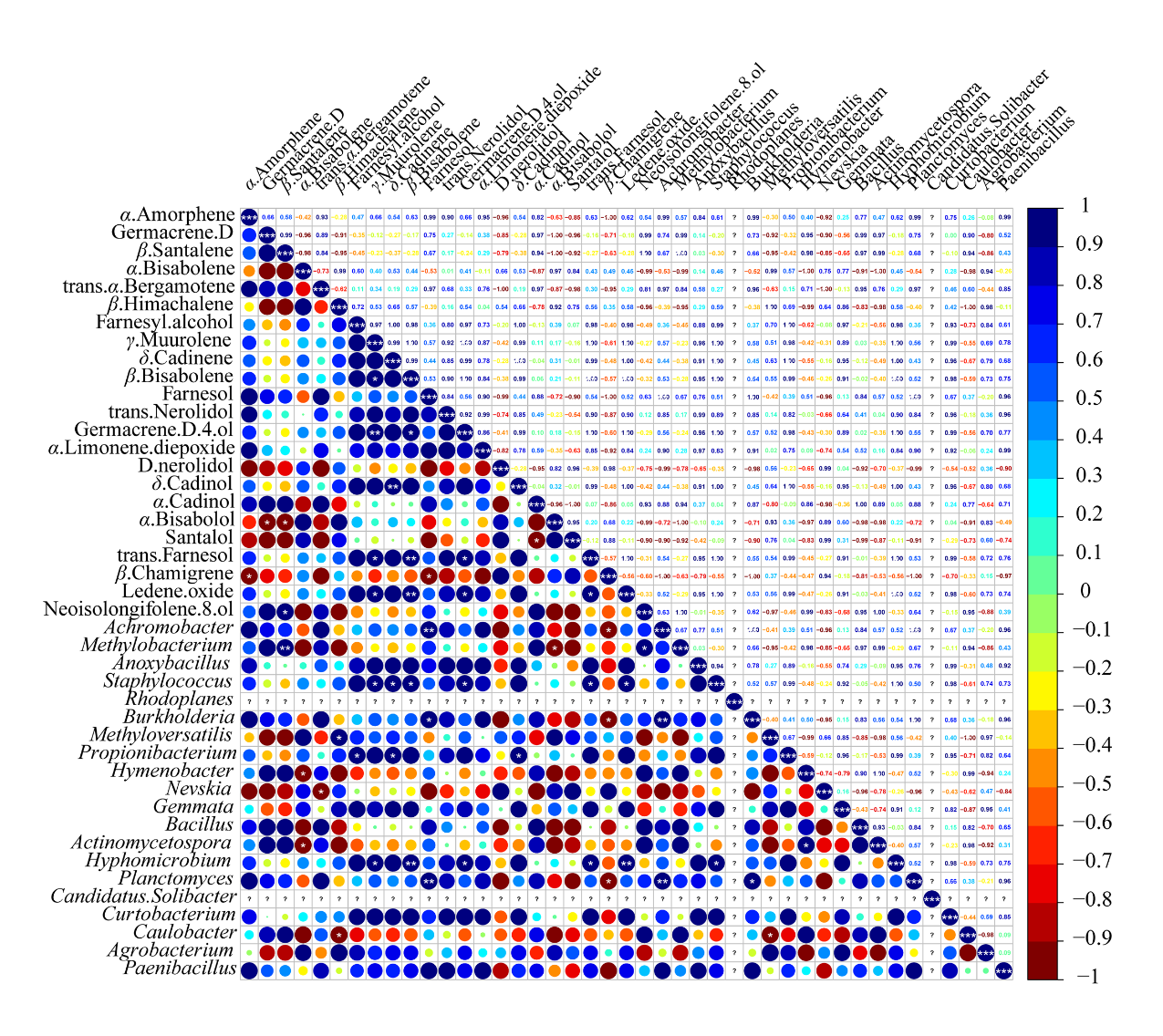


**Fig. S7.** Pearson correlations between the first 20 genera of bacterial and common chemical compositions of *S. sphenanthera.* The *, **, and *** mean significant correlation estimates at the level of 0.05, 0.01, and 0.001, respectively.


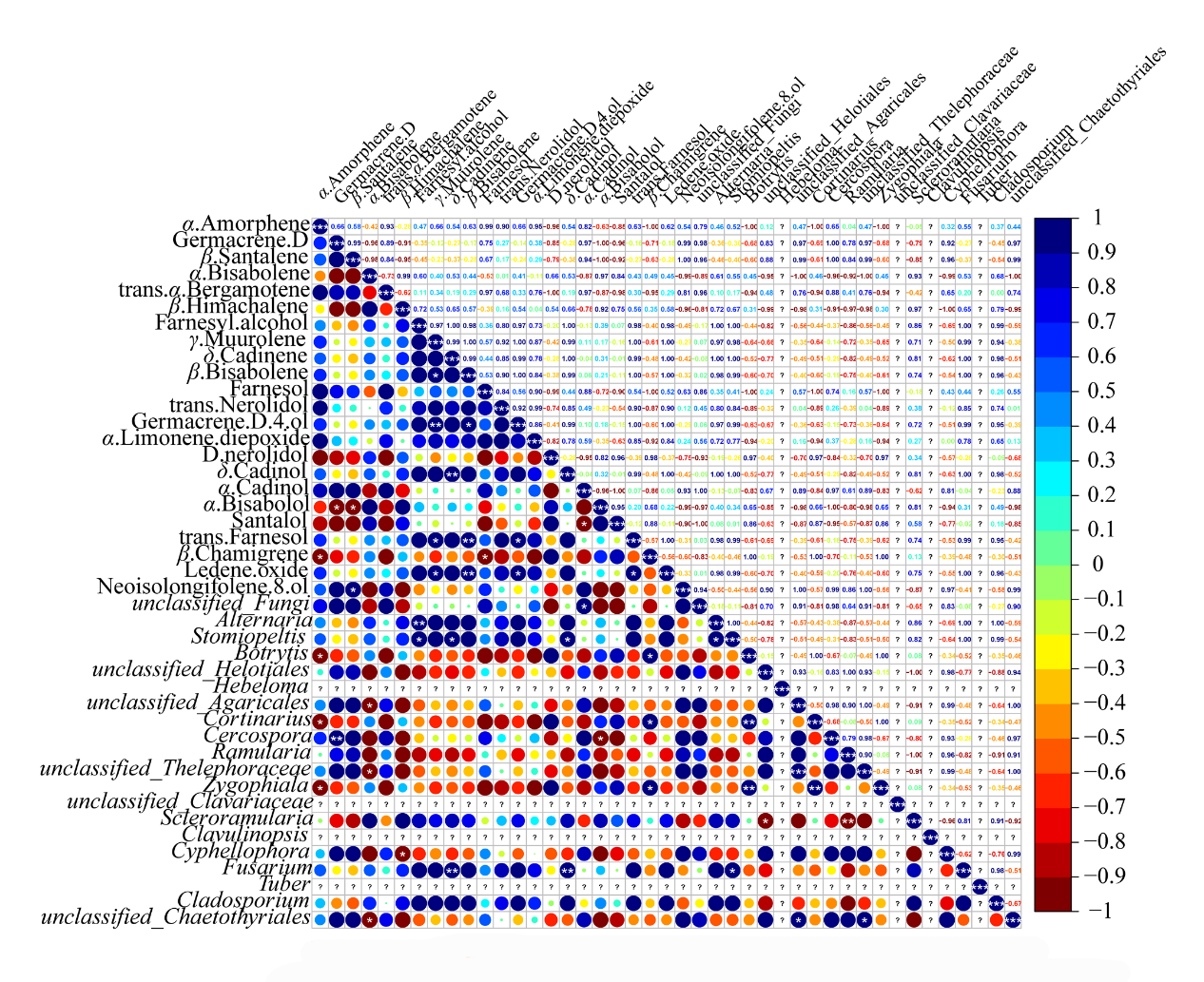


**Fig. S8.** Pearson correlations between the first 20 genera of fungal and common chemical compositions of *S. sphenanthera.* The *, **, and *** mean significant correlation estimates at the level of 0.05, 0.01, and 0.001, respectively.
